# Predictive associations between serum fatty acids and lipoproteins in healthy non-obese Norwegians: implications for cardiovascular health

**DOI:** 10.1007/s11306-015-0886-4

**Published:** 2015-11-09

**Authors:** Chenchen Lin, Tarja Rajalahti, Svein Are Mjøs, Olav Martin Kvalheim

**Affiliations:** Department of Chemistry, University of Bergen, Bergen, Norway; Fjordomics, Førde Hospital Trust, Førde, Norway; Faculty of Health Studies, Sogn og Fjordane University College, Førde, Norway

**Keywords:** Human serum, Lipoprotein subclasses, Poly-unsaturated fatty acids (PUFAs), Ratio of eicasapentaenoic acid (EPA) to arachidonic acid (AA), Docosahexaenoic acid (DHA), Selectivity ratio

## Abstract

A battery of methods for multivariate data analysis has been used to assess the associations between concentrations of fatty acids (FAs) and lipoprotein subclasses and particle size in serum for a normolipidemic population of ethnic Norwegians living in the rural Fjord region. Significant gender differences were found in the lipoprotein and FA patterns. Predictive FA patterns were revealed for lipoprotein features of importance for cardiovascular (CV) health. Thus, the subclasses of atherogenic small and very small low density lipoprotein (LDL) particles and the same subclasses of high density lipoprotein (HDL) particles were associated with a pattern of saturated FAs and mono-unsaturated C16-C18 FAs. Eicosapentaenoic acid (EPA) and the ratio of EPA to arachidonic acid (AA) had strongest associations to features that promotes CV health: (i) large average size of HDL and LDL particles, and, (ii) small average size of very low density lipoprotein (VLDL) particles. Total concentration of HDL in both genders correlated to EPA, but docosahexaenoic acid (DHA) correlated just as strongly for women. For men, docosapentaenoic acid (DPA) showed stronger association to HDL concentration than EPA. For both genders, concentration of large LDL particles showed associations to levels of EPA, but stronger to DHA and DPA. High values of EPA/AA seem to be the strongest single biomarker for good CV health in both men and women.

**Electronic supplementary material:**

The online version of this article (doi:10.1007/s11306-015-0886-4) contains supplementary material, which is available to authorized users.

## Introduction

The favorable impact of diets rich in omega-3 fatty acids (FAs) on the risk of developing cardiovascular diseases (CVDs), was suggested by the pioneering work of Danish researchers on lipids in plasma samples from Greenlandic Eskimos (Bang et al. 1971; Dyerberg et al. 1975). They compared Eskimos living in Greenland with matching control groups of Eskimos living in Denmark and ethnic Danes and found that both triglycerides (TGs) and cholesterol (Chol) levels were lower for Eskimos living in Greenland than for the two control groups. Furthermore, the levels were similar for Eskimos living in Denmark and ethnic Danes, strongly pointing to differences in dietary habits rather than genetic factors as the main reason for differences in occurrence of CVD in the investigated populations. Later, the Danish research group published a paper that compared plasma levels of the omega-6 FA, 20:4 n−6, arachidonic acid (AA) and the omega-3 FA, 20:5 n−3, eicosapentaenoic acid (EPA) in Greenlandic Eskimos with ethnic Danes (Dyerberg et al. 1978). The analyses revealed that EPA concentration for the Eskimos was more than 10 times higher than for the Danes, while the opposite picture emerged for AA with 10 times higher levels for ethnic Danes than for Eskimos. Based on these results, they proposed the hypothesis that EPA protects against CVD. This hypothesis was strengthened by Kagawa et al. (1982) who showed that serum of elderly Kohama Islanders in Japan, which were known to have the lowest incidence of CVDs in Japan, had much higher serum EPA concentrations than mainland Japanese and that this correlated with higher concentrations of high density lipoprotein (HDL). A similar study by Torres et al. (2000) compared inhabitants from two villages on the Madeira Islands. One village had a fish intake that was 10 times higher than the other, but the incidence of CVDs was only a quarter of the other. Serum from subjects on the high-fish diet showed much higher levels of EPA and docosahexaenoic acid (DHA) and lower levels of TG and Chol than for the subjects on the low-fish diet. The Seven countries epidemiological study (Menotti et al. 1999) with more than 10,000 participants confirmed the positive impact of seafood diet on occurrence of CVD by showing that Japanese and Greek Islanders displayed the same pattern of low incidences of CVD as the Greenlandic Eskimos, and the Japanese and Madeira islanders. Excellent summaries of the connection between individual or groups of FAs and their impact on CVD risk have recently been provided by (Chowdhury et al. 2014) and Michas et al. (2014).

Controlled dietary interventions followed by measurement of FAs and lipoprotein features in serum represent another route to assess the impact of dietary intake of FAs on lipoprotein patterns. Several such studies have been carried out. Egert et al. (2009) found that EPA, DHA and α-linolenic acid (ALA) were equally effective in reducing TGs, but that only DHA significantly increased serum HDL. A review and meta-analysis by Wei and Jacobson (2011) claims that DHA increases serum levels of both low density lipoproteins (LDL) and HDL, while EPA non-significantly reduces LDL, but has no effect on HDL compared to Placebo. Neff et al. (2011) did a trial with supplementation of DHA to overweight and obese adults. They used nuclear magnetic resonance (NMR) to measure the average particle size of the major lipoprotein particles in serum. They found significant decrease in very low density lipoprotein (VLDL) particles and increase in average size of LDL and HDL particles due to increased concentration of large LDL and HDL particles and reduced concentration of small LDL particles; changes that reduce CVD risk. Kelly and Adkins (2012) reviewed effects of long chain n−3 poly-unsaturated fatty acids (PUFAs) with focus on individual effects of EPA and DHA and found that only DHA reduced small dense atherogenic LDL particles. All studies on the effects on size of VLDL, LDL and HDL from DHA supplementation agreed with the results of Neff et al. (2011), but only one out of four studies with EPA reported an increase in HDL particle size and none showed increase in LDL particle size.

Thus, different kinds of investigations have confirmed that many risk factors are lowered by diets rich in the long chain omega-3 PUFAs. As discussed above, the positive impact seems to be due to decrease in the concentrations of atherogenic lipoprotein particles and TGs, increase in average size of LDL and HDL particles, and decrease in average size and concentration of VLDL particles.

The standard lipid panel does not provide enough details to quantitatively assess effects of omega-3 FAs on the lipoprotein distribution, but methods have been developed that can provide more detailed information. Usui et al. (2002) and Okazaki et al. (2005) used high performance liquid chromatography (HPLC) to partition lipoproteins into 20 subclasses. Freedman et al. (1998) developed a proton NMR method which partitioned the distribution of lipoprotein into 10 subclasses, while Hodge et al. (2011) used factor analysis to reduce the NMR profile to a single factor representing the spectral structure and used the score on this factor together with other variables in multivariate regression. Mihaleva et al. (2014) built multivariate regression models were proton NMR spectra were calibrated on lipoprotein subclasses determined by the HPLC method cited above. These new tools work on small amounts of serum without manual procedures for physical subclass separation and have provided a better understanding of which lipoprotein subclasses are associated to atherogenicity. For instance, it has been possible to correlate small dense LDL particles to increased risk of developing CVDs (Hirayama and Miida 2012).

Although much is known about CV health and its relation to FAs and lipoprotein features, a shortcoming of many investigations is that they did not incorporate both the detailed lipoprotein and FA profiles. Thus, the dietary interventions typically focused on just one or a few FAs, typically EPA and/or DHA. Most interventions were also performed for a relatively short time span and may not adequately simulate the effects of long-time dietary habits. Therefore, we have used another approach where serum samples from non-obese healthy men and women are acquired after overnight fasting and the lipoprotein and FA concentration profiles are quantified with the aim to assess the degree of association between the profiles. Since the levels of many important FAs in serum depend on continuous dietary supply, the lipoprotein features associated with these FAs must, at least partly, be associated to dietary habits. For instance, even if the omega-3 FAs EPA and DHA theoretically can be obtained by conversion of ALA, the conversion rate is too low for efficient syntheses in the human body (Egert et al. 2009).

In this work, multivariate data-analytical methods (Rajalahti and Kvalheim 2011 and refs. therein) are used with the aim to reveal gender differences and correlating patterns in lipoproteins to FA profiles. Hierarchical cluster analysis (HCA) is used to compare the FA correlation patterns of all the lipoprotein features with the objectives to reveal similarities between lipoprotein features and to detect clusters with similar FA patterns. Cross validated regression models predicting lipoprotein features from FA patterns is developed with the aims to reveal predictive patterns, to assess the strength of the relationships between FA patterns and lipoprotein features, to rank the FAs with respect to their associations to each lipoprotein feature, and, to make inferences about their possible impact on CV health through their associations to the lipoprotein features.

## Materials and methods

### Participants

136 healthy volunteers, 69 women and 67 men that were ethnic Norwegian, were recruited among the inhabitants of a rural community in the Fjord region of Western Norway. Inclusion criteria were age 18–62 years and BMI 18.5–30.0. Exclusion criteria were pregnancy, smoking, drug abuse, use of lipid-lowering drugs and established CVD, diabetes type 2 or cancer. Mean and standard deviation for the male and female cohort, respectively, was for age 41 ± 11 and 40 ± 11, for BMI, 23.5 ± 2.5 and 25.6 ± 2.8, for waist 78 ± 8 and 92 ± 8, and, for % body fat 28.5 ± 5.3 and 19.8 ± 4.4. Bioelectrical impedance analysis (MC 180, Tanita Corp, Tokyo, Japan) was used for determination of fat mass.

### Blood sampling

Blood samples were collected between 8 and 9 a.m. after overnight fasting. Serum was obtained according to a standardized protocol consisting of the following steps: (i) Blood plasma was collected in 5 mL tubes with gel (Vakuette^®^ Serum Gel with activator, G456073). (ii) Tubes were carefully turned upside-down five times and placed vertically for coagulation. (iii) After 30 min the sample was centrifuged at 2000×*g* for 10 min. Serum was then visually inspected for residues and centrifugation was repeated if residue was present. (iv) The serum tube was kept in refrigerator at 4 °C before pipetting 0,5 ml into cryo tubes. (v) The cryo tubes were then stored at −80 °C.

### Measurement of fatty acids

200 µL serum sample was weighed into 10 mL glass tubes and water was evaporated under nitrogen, followed by adding 150 µL internal standard (triheptadecanoin, 0.4855 mg/mL). After evaporating the solvent the samples were derivatized to fatty acid methyl esters (FAME) by direct esterification in methanolic HCl at 90 °C for 2 h under nitrogen atmosphere (Meier et al. 2006). FAMEs were extracted and analyzed by gas chromatography as described in Gudbrandsen et al. (2014). The samples were run in a randomized sequence and the FAME reference mixture GLC-461 (Nu-Chek Prep, Elysian, MN, USA) was analyzed as every 10th sample. Chromatographic areas were corrected by empirical response factors calculated from the GLC-461 mixture. The amounts of FAs were thereafter quantified by means of the internal standard. The total amounts of FAs in each serum sample were converted to amounts in μg per g sample by dividing with the sample weights.

### Measurement of lipoproteins

Serum lipoproteins were analyzed on an HPLC system at Skylight Biotech (Akita, Japan) according to the procedure described by Usui et al. (2002). The analysis provides concentrations of total Chol and total TG, concentrations of 4 subclasses of Chol and TG classified as chylomicrons (CM), VLDL, LDL and HDL particles, and, concentrations of 20 subclasses of Chol and TGs with 2, 5, 6 and 7 fractions, respectively, for CM, VLDL, LDL and HDL particles. In addition, average size of VDL, LDL and HDL particles for Chol and TGs is provided.

Serum apolipoproteins A1 and B were measured by turbidimetric immunoassay using commercially available kits (Sekisui Medical co., Ltd, Tokyo, Japan).

### Data preprocessing

A dataset of 18 FAs was created to be used for multivariate and statistical analysis. This dataset includes the majority of FAs that are considered biologically important. Supplementary material 1 provides a summary of the data for both genders. Total FA (TFA) concentration and the ratio EPA/AA is also included. Systematic names and abbreviations to common names defined in text are used. See supplementary material 5 for ChEBI Ids.

Cholesterol and TG for the four subclasses CM, VLDL, LDL and HDL were combined to obtain four subclasses corresponding to total concentrations of CM, VLDL, LDL and HDL particles. Similarly, Chol and TG concentrations were added for the partition of lipoproteins into 20 subclasses. The 20 subclasses were further reduced to 13 subclasses by joining the two subclasses of CM, joining the two subclasses with largest VLDL particles, joining the three subclasses of LDL particles with smallest size, joining the two subclasses of HDL with largest particles, and joining the two subclasses of HDL with smallest particle size. We then obtained one subclass for CM, four subclasses for VLDL and for LDL, and four for HDL particles, labeled as CM, VLDL-VL, VLDL-L. VLDL-M, VLDL-S, LDL-L, LDL-M, LDL-S, LDL-VS, HDL-VL, HDL-L, HDL-M, HDL-S and HDL-VS. The abbreviations VL, L, M, S and VS denote very large, large, medium, small and very small particles. The selection of subclasses to join was based on the degree of correlation between the subclasses in both men and women and the absolute concentration of particles in the various subclasses. Univariate measures for these subclasses are provided in supplementary material 2 together with total Chol and total TG concentrations and average size of VLDL, LDL and HDL particles calculated as the mean of their size in the Chol and TG fractions weighted by the corresponding fractions.

### Data analysis

Our data analysis consists of three main steps: (i) univariate analysis, (ii) exploratory multivariate analysis, and, (iii) confirmatory multivariate analysis. As some of the measured variables deviated appreciably from normality, the rank sum Wilcoxon–Mann–Whitney (WMW) nonparametric test (Wilcoxon 1945; Mann & Whitney 1947) were used for univariate comparison of men and women for the FAs (supplementary material 1) and lipoprotein features (supplementary material 2) after correcting for multiple testing by both the Bonferroni procedure and the false discovery rate (FDR) of Benjamini and Hochberg (1995). The null hypothesis was that the medians were identical for the two genders. Matlab R2013b was used for the calculations. Multivariate analyses were subsequently performed by means of Sirius Version 10.0 (Pattern Recognition Systems AS, Bergen, Norway). Prior to multivariate analysis, all variables/features were centered and standardized to unit variance. Most of the multivariate methods used in this work are described in a tutorial review by Rajalahti and Kvalheim (2011), but a summary of main procedures are provided in this section. An example of our strategy used for finding diagnostic patterns in data from clinical proteomics is described by Rajalahti et al. (2010).

The univariate analysis revealed significant gender differences and was followed by partial least squares discriminant analysis (PLS-DA) (Sjöström et al. 1986) with gender as response variable and the FA profiles and lipoproteins as input. Repeated double cross validation (RDCV), using the algorithm of Westerhuis et al. (2008) with minor modifications, was performed for optimizing the predictive ability of the FA and lipoprotein gender models. For each model, 500 repetitions were performed to calculate an average RMSEP (Martens and Dardenne 1998) and a confidence interval of two standard deviations around each PLS component. The optimal model complexity (number of PLS components to be used) was determined by comparing the RMSEP plus two standard deviations of component *a* + *1* with the RMSEP of component *a* starting with *a* = 0. If the adjusted value for component *a* + *1* was lower than RMSEP for component *a*, the procedure was repeated by comparing component *a* + *2* with component *a* + *1*. A single predictive multivariate signature, for each of the two cross-validated PLS models, was subsequently obtained by post-processing by target projection (TP) (Kvalheim and Karstang 1989). A PLS model can be described by the regression vector. By projecting the matrix of subjects time variables (**X**) on the normalized regression vector, a vector of scores proportional to the vector of predicted *y* (in this case the gender variable with zeros and ones) is obtained. A second projection, i.e. projecting the matrix of variables time subjects onto this score vector, provides the variable loadings on a single predictive component. From this we can find the most important variables for discriminating genders by calculating a selectivity ratio (SR) for each FA/lipoprotein feature. Thus, the SRs are calculated from the predictive TP signatures and given a plus or minus signs in accordance with the sign of their loadings in the TP component (Rajalahti et al. 2009a). Confidence intervals of two standard deviations are calculated from the RDCV procedure. SR plots are used to visualize the importance of individual FAs and lipoprotein features for the PLS-DA models. A supplementary measure of significance was calculated based on the concept of correct classification rate (CCR). In our previous work, this was just the percentage of correctly classified subjects as a function of the SR value (Rajalahti et al. 2009b). This approach has been modified to obtain a rank sum classification rate (RSCR) for the variables at different SR levels by a procedure calculating rank sums similar to the one used in WMW rank sum test for unpaired measurements of two groups. Using RSCR, subjects that are wrongly classified at the borderline between two groups of subjects reduce the classification rate less than subjects located far into the opposite group. This is logical since borderline subjects are less deteriorative for the discriminatory ability of a feature than strongly deviating subjects. Furthermore, one can calculate a *p* value corresponding to a particular classification rate or variable using the WMW test for a single test or modified as explained above in case of multiple testing.

After calculating FA and lipoprotein gender models, unsupervised principal component analysis (PCA) (Jolliffe 1986) was used to reveal the major correlation patterns between the lipoprotein and FA profiles for each gender. Thereafter, cross correlations between lipoprotein features and FAs for both genders were estimated as Pearson´s correlation coefficients (supplementary material 3 (women) and 4 (men)) and the FA correlation patterns were used for agglomerative HCA of lipoprotein features for both genders with Euclidean distance as metric and average linkage for clustering (Kaufman and Rousseeuw 1990).

In order to quantify the strength of association between lipoprotein features and FAs, the 24 lipoprotein features shown in supplementary material 2 were tested separately for their predictive associations to the FAs for both genders by partial least squares (PLS) regression (Wold et al. 1984). As for the gender models described above, RDCV, TP and calculation of SRs were performed and the SRs were used to rank the FAs with respect to their importance in each lipoprotein model. A summary of these lipoprotein models are provided as supplementary material 5 (women) and 6 (men). SR profiles for all the lipoprotein models are provided in supplementary material 7 (women) and 8 (men).

## Results and discussions

### Univariate assessment of lipoprotein and FA gender differences

Wilcoxon–Mann–Whitney (WMW) rank sum test (Wilcoxon 1945; Mann and Whitney 1947) was used to calculate univariate p-values for comparing the median concentrations of FAs and lipoprotein features for men and women. The null hypothesis was identical medians and the Bonferroni correction for multiple testing was subsequently applied by assuming that the FAs and the lipoproteins could be considered as two families of tests since they belong to different classes of molecules and are also measured independently by different instrumental procedures. After Bonferroni correction, serum concentrations show significant gender differences (Supplementary material 1) for 16:1 n−9 (q < 0.00005)), 18:1 n−9 (q < 0.0002), ALA (q < 0.0.003) and DPA (q < 0.04), all being higher for men than women. By using FDR as developed by Benjamini and Hochberg (1995) for comparing gender on the 20 tests for FAs, a value of p = 0.0075 from the WMW test is found sufficient for significance level of 0.025 in the multiple testing and 18:1 n−7 and TFA are found to have significantly higher concentration in serum of men in addition to those already found by the more conservative Bonferroni test. For the essential FAs, DHA, EPA and AA and the ratio EPA/AA, gender differences are not significant in the analyzed cohort.

Also the lipoprotein features (Supplementary material 2) reveal systematic gender differences. After Bonferroni correction, average size and concentration of VLDL is higher in men than in women (q < 0.000001) and so is the concentrations of CM, VLDL-VL, VLDL-L, TG, all with q < 0.000001, and, VLDL-M (q < 00003) and apolipoprotein B (apoB) with q < 0.01. Average size and concentration of HDL is higher in women than in men as is the concentrations of HDL-VL, HDL-L and HDL-M (all subclasses has q < 0.000001). Average size of LDL particles is higher in women than in men (q = 0.01), while the concentrations of the atherogenic subclasses LDL-S (q = 0.0005) and LDL-VS (q = 0.002) and the suspected atherogenic subclasses (Freedman et al. 1998) HDL-S (q = 0.0065) and HDL-VS (q < 0.002) are higher in men than in women. Calculation of FDR for a significance level of 0.01 for the multiple testing of the 24 lipoprotein features, provides the corresponding limit as p = 0.00875 for the WMW test. By this approach also the median concentrations of LDL and the subclass LDL-M are significantly different leaving only median concentrations of Chol, the subclasses VLDL-S and LDL-L as statistically indistinguishable between genders. Systematic gender differences in serum lipoprotein features have also been observed by Furusyo et al. (2013). They found, *e.g.*, significantly higher levels of CM and VLDL subclasses in Japanese men and higher levels of the subclasses of very large, large and medium HDL particles for women.

### Multivariate assessment of lipoprotein and FA gender differences

The multivariate approach described in Sect. 2.6 is now used to reveal lipoprotein and FA patterns related to gender differences.

**Fig. 1:**
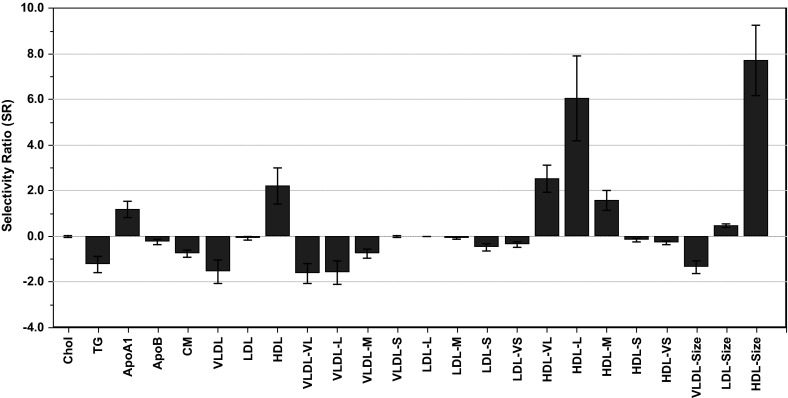
Selectivity ratio (SR) plot showing gender differences in lipoprotein patterns. Features with positive sign on SR are higher in women, while features with negative signs on the SRs are higher in men. The confidence limits around each feature are obtained from RDCV

PLS-DA accounted for 38.2 % of the gender variable (men = 0, women = 1) with lipoprotein features as input using RDCV by testing each PLS component before inclusion as described in Sect. 2.6 in order to optimize the predictive ability of the discriminatory model. Post processing of the PLS-DA model was performed by TP to obtain a single predictive multivariate component and from this component SRs were calculated for each lipoprotein feature. The SRs were also given plus or minus signs depending on the sign of their loadings in the TP component. Figure 1 displays the SR profile with confidence limits on each lipoprotein feature corresponding to two standard deviations determined from RDCV.

The size of SR is proportional to the importance of each individual lipoprotein feature for discriminating between genders. SR = ±1 for a variable corresponds to 50 % of the variance in that variable being predictive, and 50 % left in the residual. Larger average size of the HDL particles in women is the most striking feature with SR = 7.7. This is a reflection of larger concentrations of the subclasses HDL-VL (SR = 2.5), HDL-L (6.0) and HDL-M (SR = 1.6) in women compared to men. Furthermore, the total concentrations of HDL (SR = 2.2) and ApoA1 (SR = 1.2) are also significantly higher in women than in men. Other major differences are concentrations of TG (SR = −1.2) and VLDL (SR = −1.5) and its subclasses VLDL-VL (SR = −1.6) and VLDL-L (SR = −1.6), and, the average size of VLDL particles (SR = −1.4) which are all higher in men than in women since the signs of the TP loadings and thus the SRs are negative. The ranking of the features obtained from the SR plot matches closely the results from the univariate analysis for the most discriminating variables, but has the advantages of also showing whether a discriminating variable is higher or lower in one gender and by also including confidence limits in a single graph.

We can relate the SR to the discriminatory ability of the lipoprotein features with respect to gender. This is performed by calculating the RSCR for the subjects as a function of abs(SR) as explained in Sect. 2.6.

**Fig. 2:**
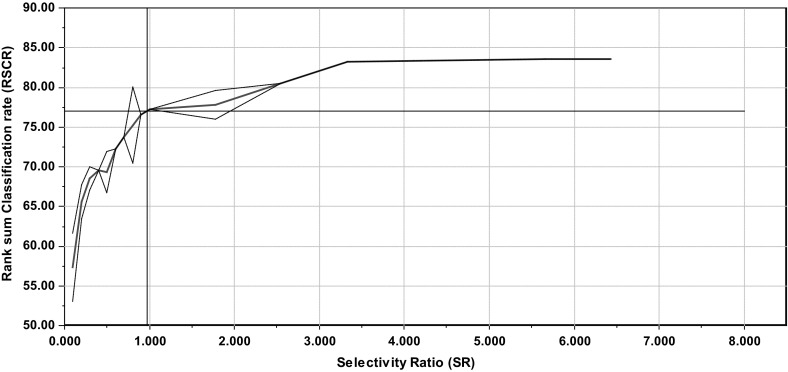
Discriminating variable (DIVA) plot obtained for gender differences in lipoprotein pattern. The *plot* shows rank sum classification rate (RSCR) as a function of increasing abs(SR). For SR = ±1 the DIVA plot shows that the RSCR is 77 %. The *dashed lines* around the *red line* showing the RSCR represent the standard deviation in intervals with more than one SR value. The RSCR is plotted in the middle of each SR interval (Color figure online)

 Figure 2 shows the RSCR for the gender differences in the lipoprotein features. With almost the same number of subjects for both genders as we have here, the expectation value of the RSCR is approx. 50 % if a variable has SR = 0 within the confidence limits. Figure 2 shows that the RSCR is 77 % for abs(SR) = 1.0 and for abs(SR) = 0.5 it is still as high as 70 %. Concentration of small (−0.5) and very small (SR = −0.4) LDL particles (see Fig. 1) are thus higher in men underlining a more atherogenic lipoprotein pattern in men compared to women (see Furusyo et al. 2013 and refs. therein). Similar to the WMW test, RSCR measures the degree of how well two groups separates on a variable, but the information content presented as a percentage is easier to comprehend than a p-value.

**Fig. 3:**
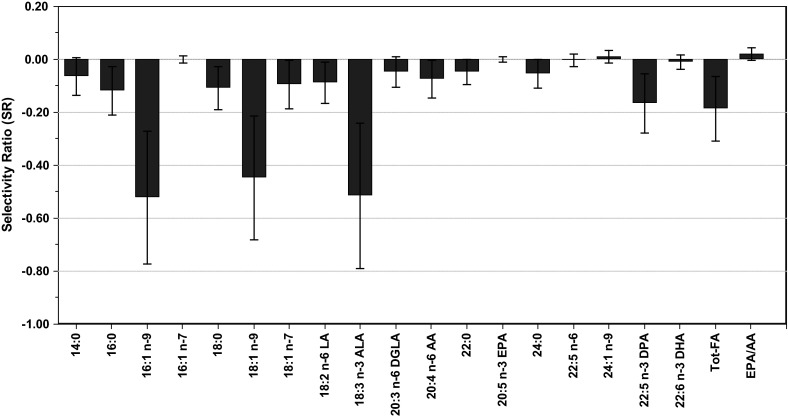
Selectivity ratio (SR) plot displaying gender differences in FA patterns. Features with positive sign on SR are higher in women, while features with negative signs on the SRs are higher in men. The confidence limit around each feature is obtained from RDCV

The cross validated PLS-DA of the FA profiles accounted for 38.8 % of the variance in the gender variable. Figure 3 shows the SR profile for the gender differences in FA profiles.

For the gender differences in FA pattern, largest differences are observed in C14-C18 FAs, i.e. 16:1 n−9 (SR = −0.52), 18:1 n−9 (SR = −0.45) and ALA (SR = −0.52) which all have RSCR higher than 70 % and Bonferroni corrected p-values less than 0.003. As they all have negative sign, their levels are higher in men than in women. The SRs for EPA, DHA, AA, and the ratio EPA/AA are all zero within the confidence limits obtained from cross validation and thus the same in men and women. Of the C20-C24 FAs, only docosapentaenoic acid (DPA) shows a gender difference.

The substantial gender differences in lipoprotein and FA patterns imply that men and women have to be treated separately in the multivariate analyses to follow.

### Correlations between lipoproteins and FAs in women

**Fig. 4:**
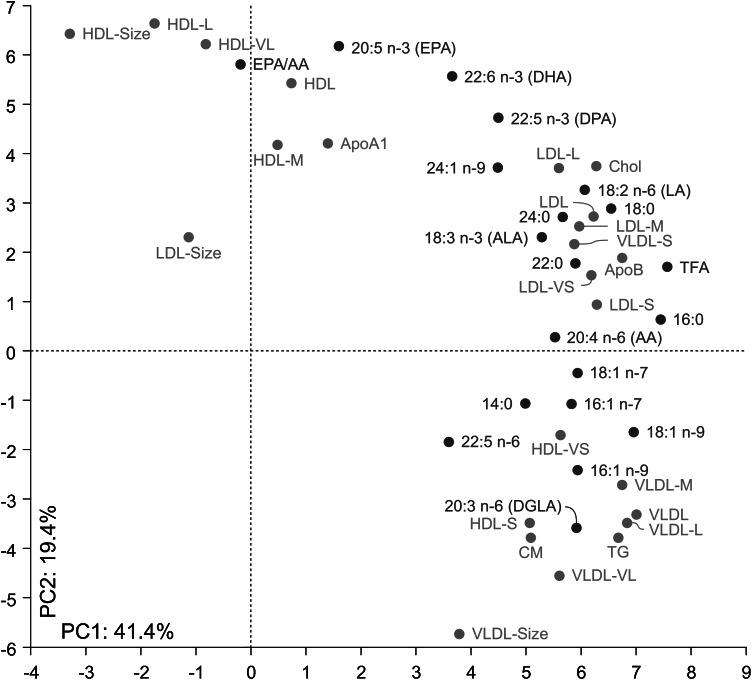
PCA loading plot of 24 lipoprotein features, 18 fatty acids, total fatty acids (TFA) and the EPA/AA ratio measured in serum for women

For the female cohort, joint PCA of the 24 lipoprotein features and 18 individual FAs, TFA and the ratio of EPA to AA showed that 60.8 % of the total variance was accounted for by the two major PCs, emphasizing a pronounced correlation between lipoproteins and FAs. Figure 4 displays the (correlation) loadings of the 44 variables on the two PCs.

Since all features are standardized to unit variance prior to PCA, the distance from origin to the location of a variable represents a quantitative measure of explained variance in that variable. Furthermore, the cosine of the angle between a pair of variables represents a quantitative measure of their explained correlation in the two-component PC model.

Most of the features related to HDL particles are located in the left upper part of the loading plot: (i) the average size of the HDL particles, (ii) the total concentration of HDL particles, and, (iii) their fractions of very large, large and medium sized HDL particles. All these features associate with good cardiovascular (CV) health. As expected, concentration of apolipoprotein A1 (ApoA1), being a major protein component of HDL particles, falls close to the cluster of the majority of HDL features. The ratio EPA/AA and concentration of EPA cluster together with size and concentrations of HDL and fractions of very large and large HDL particles. Furthermore, the concentration of EPA and the ratio EPA/AA show a negative correlation to average size of VLDL particles. According to Freedman et al. (1998) reduced size of VLDL particles reduces severity of CVD. Thus, the positions of EPA and EPA/AA in the lipoprotein correlation pattern comply with the findings of Dyerberg et al. (1978) and later confirmed by, e.g., Ninomiya et al. (2013) of the preventive effects of these two biomarkers on risk for developing CVDs.

In the lower right corner of the plot, oppositely correlated to the major part of the HDL features, characteristics related to VLDL particles appear: i) average size of VLDL particles, (ii) total concentration of VLDL, and, (iii) concentrations of fractions of very large, large and medium sized VLDL particles. As expected, concentrations of CM and TG also fall in the same region. The display shows that the concentrations of very small and small HDL particles correlate with this group of features. Freedman el al. (1998) found that a global measure of coronary artery disease (CAD) severity was positively associated with levels of large VLDL and small HDL particles.

In the upper right corner of the loading plot, all the features measured for the LDL particles cluster together with the concentration of ApoB and Chol. Correlations between HDL and LDL features are weak except for concentrations of small and very small LDL particles which correlate positively with very small HDL particles. Concentration of small LDL particles is well-known to represent a strong predictor of CVD (Hirayama and Miida 2012). Thus, small dense LDL has been accepted as a risk factor for CV events by the National Cholesterol Education Program Adult Treatment Panel III (NCEP III 2002). The correlation of small and very small HDL particles with large VLDL particles and small and very small LDL particles supports the hypothesis that these subclasses of HDL are atherogenic.

Average size of LDL particles is not well correlated to the major lipoprotein patterns of women on the two major PCs.

DHA is located in-between the HDL and LDL cluster. DHA and DPA are both strongest correlated to large LDL particles. This observation complies with previous investigations that DHA correlates positively to large LDL particles and negatively to small dense atherogenic LDL particles (Neff et al. 2011). Thus, it appears that high levels of EPA/AA, EPA, DHA and DPA correlate strongly to lipoprotein features suggestive of good CV health, but that the effects are different with EPA having strongest effect on HDL and DHA and DPA on LDL particles.

TFA is positively correlated to apoB and very small LDL particles, both being indicators of poor CV health. In summary, the loading plot provides an interpretation in line with many findings in earlier work, but shows a stronger connection between EPA and HDL properties than found previously, while DHA (and DPA) is strongly connected to LDL properties, but less connected to HDL. The FA loading pattern on PC1 and PC2 can be interpreted as a multivariate CVD risk scale with C14-18 saturated and mono-unsaturated FAs leading to increased CVD risk on one side and long chain PUFAs leading to decreased CVD risk (i.e. EPA/AA, EPA and DHA) on the other side. This observation is in line with the univariate risk scale defined by Chowdhury et al. (2014). The FA pattern also matches the lipoprotein scale representing CV health with HDL features clustering together with EPA/AA, EPA and DHA, and VLDL features and the suspected atherogenic small and very small HDL particles clustering together with the saturated and mono-unsaturated C14-C18 FAs.

### Correlations between lipoproteins and FAs in men

**Fig. 5:**
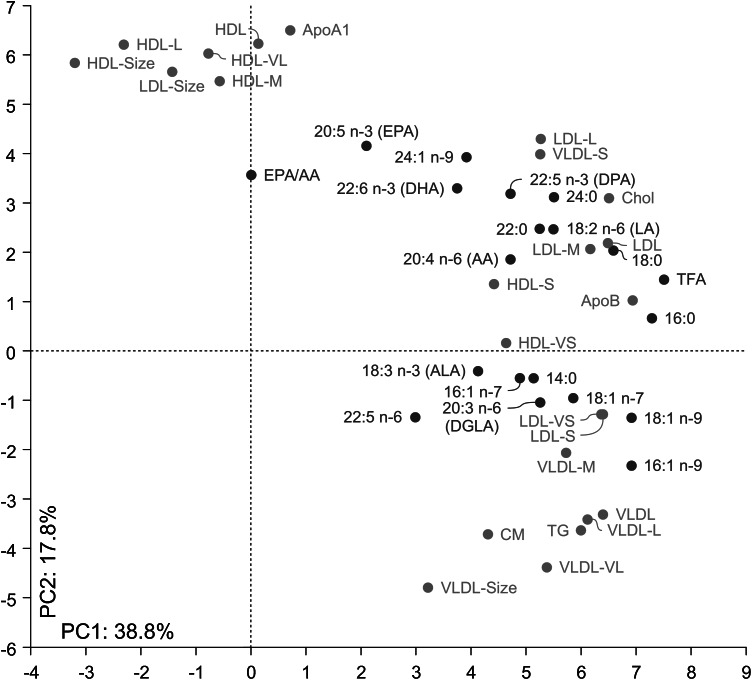
PCA loading plot of 24 lipoprotein features, 18 fatty acids, total fatty acids (TFA) and the EPA/AA ratio measured in serum for men

Figure 5 displays the loadings on the two major PCs for the lipoproteins and FAs for men.

The plot, which accounts for 56.6 % of the total variance, is similar to the loading plot for women (Fig. 4), but with some striking differences. The average size of LDL particles, which was almost uncorrelated to the major lipoprotein patterns for women, is falling in the cluster of HDL features for men and is negatively correlated to average size of VLDL particles. Thus, large average size of LDL particles is suggestive of good CV health for men. We also observe that concentration of small VLDL particles coincides with large LDL particles in men. In some methods for lipoprotein separation according to particle size, small VLDL as defined here are classified as intermediate density lipoprotein (IDL).High levels of small and very small HDL particles are associated with high levels of apoB.

We observe from Fig. 5 that the ratio EPA/AA is strongly positively correlated to average size of LDL particles and to the HDL lipoprotein features associated with good CV health. The strongest correlation is with average size of LDL particles. On the other hand, the concentrations of EPA, DHA, DPA and 24:1 n−9 are just as strongly associated with concentrations of large LDL and small VLDL particles as with the good HDL features. Concentration of TFAs correlates with apoB, total concentrations of LDL particles, and, concentrations of medium, small, and very small LDL particles. TFA also correlates with small and medium sized HDL particles. Most VLDL features cluster together and with strongest correlations to 16:1 n−9.

In summary, EPA and EPA/AA correlates strongest with good HDL features for men as for women, but the correlation is weaker for men than for women. For men, average LDL particle size correlates stronger to the favorable HDL properties than in women. EPA clusters together with DHA, DPA and nervonic acid in men, a pattern that is different from what was observed for women where EPA and the related ratio EPA/AA were more closely related to favorable HDL properties than the other long-chain PUFAs. As for women, the loading plot for men can be interpreted as indicating a multivariate CVD risk scale, but more pronounced, since the atherogenic small and very small LDL particles are located in close proximity to the VLDL features.

### Agglomerative hierarchical cluster analysis (HCA) of lipoproteins

**Fig. 6:**
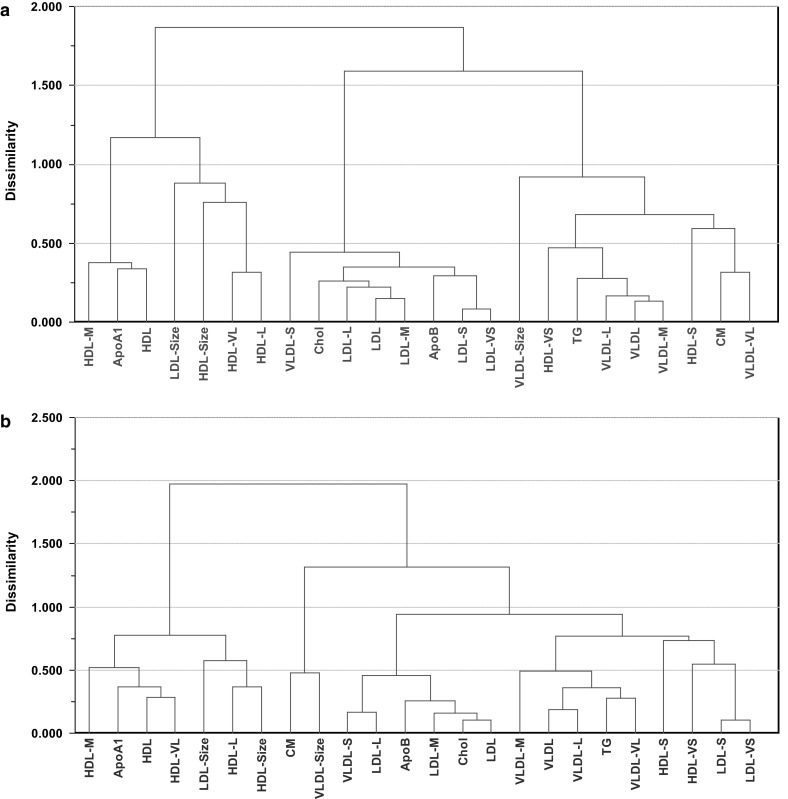
Dendrogram from agglomerative hierarchical cluster analysis calculated from average-linkage using Euclidean distance as metric. **a** women, and, **b** men

Using HCA, the similarity of the lipoprotein features for both genders based on their FA correlation patterns (supplementary material 3 (women) and 4 (men)) is assessed. The result of HCA of the lipoprotein features is presented as a dendrogram. This is a quantitative display of the similarities in FA patterns among the lipoprotein features. Men and women are treated separately. The result is displayed in Fig. 6.

For women, three main clusters are evident. ApoA1, average size of LDL and all the HDL features, except small and very small HDL particles, are connected in one cluster and all LDL features are connected in another cluster together with ApoB, Chol and small VLDL particles. In the last cluster, all the remaining VLDL features are found together with CM, TG and small and very small HDL particles. Thus, the picture revealed by HCA is identical to what was found by PCA on the two major PCs (Fig. 4).

For men, the result of HCA reveals one cluster which is identical to the one for women with most HDL features, ApoA1 and average size of LDL particles connected and a second cluster with embraces all the other lipoprotein features. Again, the overall picture from HCA is identical to the one obtained by PCA (Fig. 5). However, the second cluster splits into an LDL and a VLDL cluster located respectively in the middle and at the right end of the dendrogram which are similar to the ones for women, but the small and very small LDL particles have changed place and is located in the VLDL cluster for men with closest distance to the small and very small HDL particles.

### Predicting lipoproteins from FA patterns in women

As a last step in the analysis, we used PLS regression as a tool to validate predictive FA patterns for the lipoprotein features. Supplementary material 5 summarizes the most important characteristics for the lipoprotein regression models for women. The squared correlation coefficient (R2Y) between measured and predicted values of a lipoprotein feature represents a quantitative measure of the strength of associations between the FA patterns and the lipoprotein feature for the optimal model as determined by RDCV with confidence intervals corresponding to p = 0.05 for the PLS component selection (see Sect. 2.6). As complementary measures of predictive association between lipoprotein features and FA patterns, we have included Q2Y which measures the squared correlation between measured and predicted values for the subjects kept out from the final modelling step and subsequently predicted, and, the root mean square error of prediction (RMSEP). The most important FAs have been ranked in accordance with their strength of association to each modelled lipoprotein feature. This ranking was obtained by making a TP to separate the lipoprotein-related information carried by each FA from systematic interfering variation and then calculating the SRs (supplementary material 7). As an additional measure of strength of association to the lipoprotein features, we have included the correlation coefficients (supplementary material 3) in the raw data in parentheses for the highest ranked and lowest ranked of the most important FAs.

It should be noticed that measures of physical activity/non-activity has been shown to have a large impact on lipoprotein distribution and largest on HDL features (Aadland et al. 2013). Physical activity triggers reverse cholesterol transport (RCT) whereby Chol is transported from the fat deposit to the liver. This process increases total HDL concentration and shifts the distribution towards larger average size of HDL particles. On the other hand, a sedentary lifestyle leads to a reduction in total HDL concentration and reduction in average size of HDL. The lack of measures of physical activity in the modelling leads to a reduction in predictive power of some PLS models, but our models are developed for the purpose of strengthening the interpretation, not to substitute lipoprotein features with predictions from FA profiles.

Supplementary material 5 reveals positive predictive associations of total concentration of HDL particles to DHA and EPA in women, but also with linoleic acid (LA), 16:0, TFA and ALA. This mixed pattern is disentangled when we look at models for subclasses of HDL. Thus, strong predictive positive associations exist between concentrations of large HDL particles and EPA, DHA and DPA in that order. The strongest association is, however, with the ratio EPA/AA which also possesses the strongest positive association to average size of HDL particles. Large HDL and average size of HDL particles both shows strong negative association with 20:3 n−6, dihomo-γ-linolenic acid (DGLA), which has the strongest positive association to TG. The picture for very large HDL particles is similar to the pattern for large HDL particles, but the predictive strength is weaker. Concentrations of small and very small HDL particles have strong associations to DGLA, but also saturated and mono-unsaturated C16–C18. Total concentration of LDL is predicted by concentrations of saturated FAs, DPA and DHA, ALA and LA. Furthermore, we observe strong predictive relationships between concentration of large and medium sized LDL particles and DPA and DHA, and weak predictive relationships with EPA in women, but with some saturated FAs also showing strong associations with these lipoprotein features. Small and very small LDL particles correlate to saturated FAs. ApoB shows almost the same pattern emphasizing its connection to the atherogenic subclasses of LDL. Total concentration and size of VLDL particles and the subclasses of very large, large and medium VLDL have strong predictive associations with DGLA and C16–C18 omega-7 and omega-9 FAs. The pattern is similar to what we obtained for small and very small HDL. LA and C16–C18 omega-7 FAs have strongest association to concentration of ApoA1.

### Predicting lipoproteins from FA patterns in men

Supplementary material 6 shows R2Y, Q2Y, RMSEP and the most important predictive FAs for the lipoprotein features for men. The complete SR profiles are listed in supplementary material 8.

Average size of LDL particles are positively correlated to EPA/AA, EPA and DHA and negatively correlated to ALA and C16-C18 omega-7 and omega-9 FAs. Large average size of LDL has been shown to reduce risk of atherosclerosis so this observation is in accordance with protective effects of EPA and DHA. Total LDL is predicted by total concentration of FAs and saturated FAs, *i.e.* 16:0 and 18:0. Large LDL particles are most strongly associated to concentration of TFAs and saturated FAs, but also DHA, DPA and EPA have strong to moderate associations to large LDL particles. Small and very small LDL particles show identical predictive patterns with TFAs, saturated and omega- 9 FAs with 16 and 18 carbons possessing the strongest associations to these lipoprotein features. The same pattern is evident for apoB replicating the connection to saturated FAs that was found for women. Also very small HDL particles exhibit similar association pattern in men as in women. Very large, large and medium sized HDL particles showed no predictive FA patterns in men. These subclasses are strongly influenced by physical activity by triggering RCT and thus impacting the distribution of HDL. Total concentrations of HDL and apoA1 share weak predictive relationships to DPA, AA, and EPA. Average size of HDL particles is most strongly positively associated to EPA/AA and EPA and negatively to DGLA, just as for women, but the associations are weaker. Concentrations of very large, large and medium sized VLDL particles have similar predictive FA patterns in men and women with strongest associations to C16-C18 omega-9 FAs.

## Concluding remarks

Metabolic profiling of serum together with a battery of multivariate data-analytical methods have been used to quantify gender differences in lipoprotein and FA patterns and to reveal predictive FA patterns for lipoprotein subclasses and particle size in healthy non-obese subjects. EPA and EPA/AA were found to correlate stronger than DHA to lipoprotein features associated with good CV health, but less so for men than women. Our analyses support the hypothesis that small and very small HDL particles correlate to an unhealthy CV pattern of high serum concentrations of TGs and VLDL, large average size of VLDL particles and increased levels of atherogenic subclasses of small and very small LDL particles.

Since the level of many of the FAs in serum depends on continuous dietary input, diet is an underlying factor with a major impact on the lipoprotein pattern. However, also physical activity has a profound influence on the lipoprotein pattern (Aadland et al. 2013). Combining FA profiles with quantitative descriptors of physical activity and other factors affecting the lipoprotein distribution, may provide increased insight into how these factors play together in shaping the lipoprotein patterns.

## Electronic supplementary material

Supplementary material 1 (DOCX 14 kb)

Supplementary material 2 (DOCX 15 kb)

Supplementary material 3 (XLSX 13 kb)

Supplementary material 4 (XLSX 13 kb)

Supplementary material 5 (DOCX 17 kb)

Supplementary material 6 (DOCX 17 kb)

Supplementary material 7 (XLSX 13 kb)

Supplementary material 8 (XLSX 14 kb)

Supplementary material 9 (ODS 47 kb)

## References

[CR1] Aadland E, Andersen JR, Anderssen SA, Kvalheim OM (2013). Physical activity versus sedentary behavior: Associations with lipoprotein particle subclass concentrations in healthy adults. PLoS One.

[CR2] Bang HO, Dyerberg J, Nielsen AB (1971). Plasma lipid and lipoprotein pattern in Greenlandic West-coast Eskimos. Lancet.

[CR3] Benjamini Y, Hochberg Y (1995). Controlling the false discovery rate: A practical and powerful approach to multiple testing. Journal of the Royal Statistical Society Series B.

[CR4] Chowdhury R, Warnakula S, Kunutsor S (2014). Association of dietary, circulating, and supplement fatty acids with coronary risk—A systematic review and meta-analysis. Annals of Internal Medicine.

[CR5] Dyerberg J, Bang HO, Hjorne N (1975). Fatty acid composition of the plasma lipids in Greenland Eskimos. American Journal of Clinical Nutrition.

[CR6] Dyerberg J, Bang HO, Stoffersen E, Moncad S, Vane JR (1978). Eicosapentaenoic acid and prevention of thrombosis and atherosclerosis?. Lancet.

[CR7] Egert S, Kannenberg F, Somoza V, Erbersdobler HF, Wahrburg U (2009). Dietary a-linolenic acid, EPA, and DHA have differential effects on LDL fatty acid composition but similar effects on serum lipid profiles in normolipidemic humans. Journal of Nutrition.

[CR8] Freedman DS, Otvos JD, Jeyarajah EJ, Barboriak JJ, Anderson AJ, Walker JA (1998). Relation of lipoprotein subclasses as measured by proton nuclear magnetic resonance spectroscopy to coronary artery disease. Atheriosclerosis Thrombosis and Vascular Biology.

[CR9] Furusyo N, Ai M, Okazaki M (2013). Serum cholesterol and triglyceride reference ranges of twenty lipoprotein subclasses for healthy Japanese men and women. Atherosclerosis.

[CR10] Gudbrandsen OA, Kodama Y, Mjøs SA (2014). Effects of duodenal switch alone or in combination with sleeve gastrectomy on body weight and lipid metabolism in rats. Nutrition & Diabetes.

[CR11] Hirayama S, Miida T (2012). Small dense LDL: An emerging risk factor for cardiovascular disease. Clinica Chimica Acta.

[CR12] Hodge AM, Jenkins AJ, English DR, O’Dea K, Giles GG (2011). NMR-determined lipoprotein subclass profile is associated with dietary composition and body size. Nutrition, Metabolism & Cardiovascular Diseases.

[CR13] Jolliffe IT (1986). Principal component analysis.

[CR14] Kagawa Y, Nishizawa M, Suzuki M (1982). Eicosapolyenoic acids of serum lipids of Japanese islanders with low incidence of cardiovascular diseases. Journal of Nutritional Science and Vitaminology.

[CR15] Kaufman L, Rousseeuw PJ (1990). Finding groups in data: An introduction to cluster analysis.

[CR16] Kelley DS, Adkins Y (2012). Similarities and differences between the effects of EPA and DHA on markers of atherosclerosis in human subjects. Proceedings of the Nutrition Society.

[CR17] Kvalheim OM, Karstang TV (1989). Interpretation of latent-variable regression models. Chemometrics and Intelligent Laboratory Systems.

[CR18] Mann HB, Whitney DR (1947). On a test of whether one of two random variables is stochastically larger than the other. The Annals of Mathematical Statistics.

[CR19] Martens HA, Dardenne P (1998). Validation and verification of regression in small data sets. Chemometrics and Intelligent Laboratory Systems.

[CR20] Meier S, Mjos SA, Joensen H, Grahl-Nielsen O (2006). Validation of a one-step extraction/methylation method for determination of fatty acids and cholesterol in marine tissues. Journal of Chromatography A.

[CR21] Menotti A, Kromhout D, Blackburn H (1999). Food intake patterns and 25-year mortality from coronary heart disease: Cross-cultural correlations in the Seven Countries Study. The Seven Countries Study Research Group. European Journal of Epidemiology.

[CR22] Michas M, Micha R, Zampelas A (2014). Dietary fats and cardiovascular disease: Putting together the pieces of a complicated puzzle. Atherosclerosis.

[CR23] Mihaleva VV, van Schalkwijk DB, de Graaf AA (2014). A systematic approach to obtain validated partial least square models for predicting lipoprotein subclasses from serum NMR spectra. Analytical Chemistry.

[CR24] Neff LM, Culiner J, Cunningham-Rundles S (2011). Algal docosahexaenoic acid affects plasma lipoprotein particle size distribution in overweight and obese adults. Journal of Nutrition.

[CR25] Ninomiya T, Nagata M, Hata J (2013). Association between ratio of serum eicosapentaenoic acid to arachidonic acid and risk of cardiovascular disease: The Hisayama study. Atherosclerosis.

[CR26] Okazaki M, Usui S, Ishigami M (2005). Identification of unique lipoprotein subclasses for visceral obesity by component analysis of cholesterol profile in high-performance liquid chromatography. Arteriosclerosis, Thrombosis, and Vascular Biology.

[CR27] Rajalahti T, Arneberg R, Berven FS, Myhr K-M, Ulvik RJ, Kvalheim OM (2009). Biomarker discovery in mass spectral profiles by means of selectivity ratio plot. Chemometrics and Intelligent Laboratory Systems.

[CR28] Rajalahti T, Arneberg R, Kroksveen AC, Berle M, Myhr K-M, Kvalheim OM (2009). Discriminating variables test and selectivity ratio plot—Quantitative tools for interpretation and variable (biomarker) selection in complex spectral or chromatographic profiles. Analytical Chemistry.

[CR29] Rajalahti T, Kroksveen AC, Arneberg R, Berven FS, Vedeler C, Myhr K-M, Kvalheim OM (2010). A multivariate approach to reveal biomarker signatures for disease classification: Application to mass spectral profiles of cerebrospinal fluid from patients with multiple sclerosis. Journal of Proteome Research.

[CR30] Rajalahti T, Kvalheim OM (2011). Multivariate data analysis in pharmaceutics: A tutorial review. International Journal Pharmaceutics.

[CR31] Sjöström M, Wold S, Söderström B, Gelsema ES, Kanal LN (1986). Pattern recognition in practice II.

[CR32] Third Report of the National Cholesterol Education Program (NCEP) Expert Panel on Detection, Evaluation, and Treatment of High Blood Cholesterol in Adults (Adult Treatment Panel III) final report. (2002). National Cholesterol Education Program (NCEP) Expert Panel on Detection, Evaluation, and Treatment of High Blood Cholesterol in Adults (Adult Treatment Panel III). *Circulation, 106*, 3143–3421. 12485966

[CR33] Torres IC, Mira L, Ornelas CP, Melim A (2000). Study of the effects of dietary fish intake on serum lipids and lipoproteins in two populations with different dietary habits. British Journal of Nutrition.

[CR34] Usui S, Hara Y, Hosaki S, Okazaki M (2002). A new on-line dual enzymatic method for simultaneous quantification of cholesterol and triglycerides in lipoproteins by HPLC. Journal of Lipid Research.

[CR35] Wei MY, Jacobson TA (2011). Effects of eicosapentaenoic acid versus docosahexaenoic acid on serum lipids: A systematic review and meta-analysis. Current Atherosclerosis Reports.

[CR36] Westerhuis JA, Hoefsloot HCJ, Smit S (2008). Assessment of PLSDA cross validation. Metabolomics.

[CR37] Wilcoxon F (1945). Individual comparisons by ranking methods. Biometrics Bulletin.

[CR38] Wold S, Ruhe A, Wold H, Dunn WJ (1984). The collinearity problem in linear regression. The partial least squares (PLS) approach to generalized inverses. SIAM Journal on Scientific and Statistical Computing.

