# Supplementary material for: Predictive associations between serum fatty acids and lipoproteins in healthy non-obese Norwegians: implications for cardiovascular health
Source: Metabolomics. 2015 Nov 9;12:6. doi: 10.1007/s11306-015-0886-4 (PMC4639572; doi:10.1007/s11306-015-0886-4)
Supplement: Supplementary file 1 — Supplementary material 1 (DOCX 14 kb) [file 11306_2015_886_MOESM1_ESM.docx]

Supplementary material 1A. Univariate statistical measures calculated for fatty acids for the female and male cohorts. Median, min and max values are given in units of μg per g sample. p_WMW_ are the p-values calculated from the nonparametric Wilcoxon-Mann-Whitney (WMW) rank sum test (Wilcoxon 1945; Mann and Whitney 1947), while p_Bonferroni_ are the values after correcting for multiple testing regarding all the fatty acid variables as one family of tests.

Women (N=69) Men (N=67)

Variable Median Min Max Median Min Max p_WMW_ q=p_Bonferroni_

**14:0** 37.8 13.1 93.3 40.9 19.5 138.2 0.036287 0.725743

**16:0** 839.6 534.8 1343.5 907.2 548.8 1668.2 0.018529 0.370591

**16:1 n-9** 13.6 5.1 30.4 17.3 8.9 36.0 2.3*10^-6^ 0.000047

**16:1 n-7** 72.1 23.1 196.9 67.5 27.9 183.5 0.889221 1

**18:0** 283.3 175.9 467.1 304.2 196.5 451.9 0.040775 0.815493

**18:1 n-9** 708.3 455.8 1375.4 896.1 434.8 1562.6 9.2*10^-6^ 0.000184

**18:1 n-7** 53.2 31.3 117.0 60.5 36.4 94.4 0.006518 0.130367

**18:2 n-6 (LA)** 1214.7 664.9 1850.7 1248.4 651.6 1942.5 0.238156 1

**18:3 n-3 (ALA)** 26.4 7.7 54.7 34.9 13.4 74.3 0.000142 0.002844

**20:3 n-6 (DGLA)** 54.4 19.8 125.0 60.1 33.0 111.9 0.027938 0.558751

**20:4 n-6 (AA)** 244.7 133.6 438.0 279.8 126.6 516.0 0.060648 1

**22:0** 36.8 20.1 65.0 37.6 21.4 74.9 0.410691 1

**20:5 n-3 (EPA)** 52.3 13.9 261.8 55.6 13.0 209.8 0.415660 1

**24:0** 36.6 19.0 72.5 38.6 20.3 74.6 0.206837 1

**22:5 n-6** 4.2 0.0 18.3 5.0 2.2 13.2 0.168982 1

**24:1 n-9** 63.4 35.0 108.3 63.1 38.8 102.0 0.571487 1

**22:5 n-3 (DPA)** 32.0 20.3 56.4 35.4 20.6 66.3 0.001912 0.038243

**22:6 n-3 (DHA)** 118.1 58.9 319.1 120.6 62.5 310.1 0.428239 1

**TFA** 3896.3 2403.7 6236.3 4284.5 2607.7 6497.6 0.005411 0.108238

**EPA/AA** 0.208 0.051 0.937 0.225 0.048 0.798 0.934088 1
