# Supplementary material for: Predictive associations between serum fatty acids and lipoproteins in healthy non-obese Norwegians: implications for cardiovascular health
Source: Metabolomics. 2015 Nov 9;12:6. doi: 10.1007/s11306-015-0886-4 (PMC4639572; doi:10.1007/s11306-015-0886-4)
Supplement: Supplementary file 2 — Supplementary material 2 (DOCX 15 kb) [file 11306_2015_886_MOESM2_ESM.docx]

Supplementary material 1B. Mean and standard deviation (SD) for lipoprotein features for the female and male cohorts. Median, min and max concentrations are given in units of mg per dl serum, while particle size is given as nm. p_WMW_ are the p-values calculated from the nonparametric Wilcoxon-Mann-Whitney (WMW) rank sum test (Wilcoxon 1945; Mann and Whitney 1947), while p_Bonferroni_ are the values after correcting for multiple testing regarding all the lipoprotein variables as one family of tests.

Women (N=69) Men (N=67)

Variable Median Min Max Median Min Max p_WMW_ q=p_Bonferroni_

**Chol** 176.2 130.1 295.9 196.9 97.1 267.7 0.043415 1

**TG** 63.4 32.8 183.1 96.4 43.4 227.6 4.3*10^-7^ 0.000010

**CM** 1.36 0.03 14.66 3.58 0.30 31.54 8.6*10^-9^ 2.1*10^-7^

**VLDL** 63.4 22.8 206.9 100.4 37.0 229.5 5.5*10^-9^ 1.3*10^-7^

**LDL** 109.2 74.0 208.4 134.6 66.0 198.9 0.003796 0.091110

**HDL** 69.5 37.0 104.6 55.3 34.1 80.0 8.3*10^-10^ 2.0*10^-8^

**VLDL-VL** 10.5 0.7 79.6 26.6 3.0 89.2 1.8*10^-9^ 4.4*10^-8^

**VLDL-L** 21.4 5.7 66.4 37.1 10.5 83.2 1.6*10^-8^ 4.0*10^-7^

**VLDL-M** 15.9 4.3 41.0 21.4 10.8 45.3 0.000011 0.000259

**VLDL-S** 14.2 7.4 28.9 15.8 7.9 28.1 0.025838 0.620122

**LDL-L** 39.7 21.5 76.8 43.6 20.3 70.5 0.178621 1

**LDL-M** 47.4 30.4 94.2 58.3 26.7 91.1 0.004124 0.098984

**LDL-S** 16.6 9.4 37.3 22.1 11.1 37.5 0.000021 0.000502

**LDL-VS** 6.65 4.34 13.4 8.58 4.90 15.5 0.000087 0.002089

**HDL-VL** 4.09 1.54 9.63 2.43 1.19 5.23 2.4*10^-11^ 5.7*10^-10^

**HDL-L** 17.5 2.8 40.2 8.1 0.6 23.1 1.5*10^-11^ 3.5*10^-10^

**HDL-M** 25.1 11.8 34.2 19.9 10.9 31.5 6.1.*10^-9^ 1.5*10^--7^

**HDL-S** 15.8 11.4 25.6 17.8 13.7 25.1 0.000269 0.006460

**HDL-VS** 6.25 3.43 9.69 7.02 5.03 9.80 0.000083 0.001997

**VLDL-Size** 42.8 37.9 48.9 45.6 40.2 51.6 1.1*10^-8^ 2.7*10^-7^

**LDL-Size** 26.0 25.46 26.43 25.9 25.22 26.34 0.000422 0.010132

**HDL-Size** 11.02 10.34 11.41 10.63 10.26 11.12 2.9*10^-12^ 7.0*10^-11^

**ApoA1** 148.0 91.6 182.0 134.0 95.2 169.0 3.2*10^--7^ 0.000008

**ApoB** 82.0 54.000 142.0 98.6 49.6 136.3 0.000411 0.009875
