# Supplementary material for: Predictive associations between serum fatty acids and lipoproteins in healthy non-obese Norwegians: implications for cardiovascular health
Source: Metabolomics. 2015 Nov 9;12:6. doi: 10.1007/s11306-015-0886-4 (PMC4639572; doi:10.1007/s11306-015-0886-4)
Supplement: Supplementary file 5 — Supplementary material 5 (DOCX 17 kb) [file 11306_2015_886_MOESM5_ESM.docx]

Supplementary material 3A. Lipoprotein features modelled on the basis of fatty acid profiles for women (N=69).

Variable R2Y^1^ Q2Y^2^ RMSEP^3^ FAs ranked after importance in model

**Chol** 0.72 0.52 28 LA (0.70)^4^, DPA, TFA, 18:0, 24:0, 16:0, DHA, ALA (0.57)

**TG** 0.71 0.60 19 DGLA (0.73), 16:1 n-7, 16:1 n-9, 18:1 n-9, 16:0 (0.71)

**CM** 0.64 0.43 1.7 14:0 (0.73), DGLA, 16:0, 16:1 n-9, 18:1 n-9 (0.54)

**VLDL** 0.59 0.49 23 18:1 n-9 (0.75), DGLA, 16:0, 16:1 n-9, TFA, 16:1 n-7 (0.62)

**LDL** 0.50 0.41 26 18:0 (0.60), DPA, TFA, LA, DHA, 24:0, ALA (0.52)

**HDL** 0.52 0.19 11 LA (0.35), 16:0, DHA, EPA, TFA, ALA (0.25)

**VLDL-VL** 0.62 0.39 9.7 DGLA (0.65), 14:0, 16:1 n-7, 16:1 n-9, 18:1 n-9, 16:0 (0.54)

**VLDL-L** 0.56 0.49 8.7 18:1 n-9 (0.76), DGLA, 16:0, 16:1 n-9, TFA, 16:1 n-7 (0.61)

**VLDL-M** 0.54 0.45 5.5 18:1 n-9 (0.72), 16:0, 16:1 n-9, TFA (0.63)

**VLDL-S** 0.40 0.34 3.9 TFA (0.62), 16:0, 18:0, 18:1 n-9, LA (0.46)

**LDL-L** 0.51 0.41 9.0 DPA (0.62), 18:0, DHA, TFA, LA, 24:0, 24:1 n-9 (0.48), EPA (0.29)

**LDL-M** 0.47 0.37 12 DPA (0.59), 18:0, DHA, LA, TFA, 24:0 (0.52), EPA (0.22)

**LDL-S** 0.35 0.30 5.0 TFA (0.59), 16:0, 18:0, LA, 24:0, 22:0 (0.48)

**LDL-VS** 0.36 0.32 1.8 TFA (0.60), 16:0, 18:0, LA, 24:0, 22:0 (0.49)

**HDL-VL** 0.26 0.16 1.5 EPA/AA (0.43), EPA, DHA (0.37), DPA (0.29)

**HDL-L** 0.52 0.24 7.0 EPA/AA (0.41), EPA, DGLA (-0.43), DHA (0.32)

**HDL-M** No predictive model

**HDL-S** 0.68 0.59 2.0 DGLA (0.76), 16:1 n-7, 16:1 n-9, 18:1 n-9, 18:1 n-7, 16:0 (0.59)

**HDL-VS** 0.50 0.42 1-0 18:1 n-9 (0.63), DGLA, 16:1 n-9, 16:0, 18:1 n-7, 16:1 n-7 (0.58)

**VLDL-Size** 0.60 0.42 1.7 DGLA (0.61), 16:1 n-9, 18:1 n-9, 16:1 n-7, 14:0, EPA/AA (-0.48))

**LDL-Size** No predictive model

**HDL-Size** 0.36 0.29 0.22 EPA/AA (0.44), EPA (0.41), DGLA (-0.58)

**ApoA1** 0.53 0.22 15 LA (0.40), 18:1 n-7, 16:1 n-7, 16:0, TFA (0.32)

**ApoB** 0.46 0.42 16 TFA (0.67), 18:0, 16:0, LA, 24:0, 22:0 (0.58)

^1^R2Y implies the squared Pearson correlation coefficient calculated between modelled and measured values for the lipoprotein features.

^2^Q2Y implies the squared Pearson correlation coefficient calculated between predicted and measured values for the lipoprotein features.

^3^RMSEP implies the root mean square error of prediction (Martens and Dardenne 1998) estimated from repeated double cross validation (Westerhuis et al. 2008).

^4^Pearson´s correlation coefficient estimated from the raw data, see suppl. material. 2A.
