# Supplementary material for: Predictive associations between serum fatty acids and lipoproteins in healthy non-obese Norwegians: implications for cardiovascular health
Source: Metabolomics. 2015 Nov 9;12:6. doi: 10.1007/s11306-015-0886-4 (PMC4639572; doi:10.1007/s11306-015-0886-4)
Supplement: Supplementary file 6 — Supplementary material 6 (DOCX 17 kb) [file 11306_2015_886_MOESM6_ESM.docx]

Supplementary material 3B. Lipoprotein features modelled on the basis of fatty acid profiles for men (N=67).

Variable R2Y^1^ Q2Y^2^ RMSEP^3^ FAs ranked after importance in model

**Chol**  0.50 0.46 27 TFA (0.71)^4^, 16:0, 18:0, LA, 24:0, 22:0 (0.64)

**TG** 0.85 0.76 21 16:1 n-9 (0.80), 18:1 n-9, 16:0, 14:0, 18:1 n-7, 16:1 n-7 (0.57)

**CM** 0.63 0.46 4.4 14:0 (0.65), 16:1 n-7, 16:1 n-9, 16:0 (0.56)

**VLDL** 0.82 0.69 23 16:1 n-9 (0.80), 18:1 n-9, 16:0, TFA, 18:1 n-7 (0.60)

**LDL** 0.47 0.43 22 TFA (0.69), 16:0, 18:0, 18:1 n-9 (0.55)

**HDL** 0.43 0.15 9.1 DPA (0.27), 18:0, AA, EPA (0.18)

**VLDL-VL** 0.79 0.66 12 16:1 n-9 (0.74), 18:1 n-9, 16:0, 14:0, 16:1 n-7, 18:1 n-7 (0.56)

**VLDL-L** 0.76 0.62 9.4 16:1 n-9 (0.75), 18:1 n-9, TFA, 16:0 , 18:1 n-7 (0.59)

**VLDL-M** 0.71 0.54 5.3 16:1 n-9 (0.71), 18:1 n-9, TFA, 18:1 n-7, ALA (0.51)

**VLDL-S** 0.41 0.37 3.6 TFA (0.61), 16:0, 18:0, 18:1 n-9, 24:0, LA (0.50)

**LDL-L** 0.48 0.36 8.8 24:0 (0.66), TFA, 22:0, 18:0, LA, 24:1 n-9, DHA, DPA, EPA (0.38)

**LDL-M** 0.43 0.38 10 TFA (0.65), 16:0, 18:0, 18:1 n-9, 16:1 n-9 (0.47)

**LDL-S** 0.40 0.36 4.7 TFA (0.62), 16:0, 18:0, 18:1 n-9, 16:1 n-9 (0.59)

**LDL-VS** 0.41 0.37 1.8 TFA (0.63), 16:0, 18:0, 18:1 n-9, 16:1 n-9 (0.59)

**HDL-VL/L/M** No predictive model

**HDL-S** 0.26 0.20 2.2 16:0 (0.53), TFA, 18:0, 16:1 n-9, 18:1 n-9, 18:1 n-7 (0.23)

**HDL-VS** 0.26 0.20 1.0 TFA (0.46), 16:0, 18:0, 18:1 n-9, 16:1 n-9 (0.39)

**VLDL-Size** 0.47 0.38 2.1 18:1 n-7 (0.37), 16:1 n-9, DGLA, 14:0, EPA/AA (-0.29)

**LDL-Size**  0.28 0.15 0.22 EPA/AA (0.34), EPA (0.31), DHA (0.19)

**HDL-Size** 0.25 0.11 0.21 EPA/AA (0.21), EPA, DGLA (-), 16:1 n-9 (-), 18:1 n-9 (-0.37)

**ApoA1** 0.50 0.29 14 DPA (0.31), 18:0, AA, EPA (0.25)

**ApoB** 0.52 0.49 14 TFA (0.72), 16:0, 18:0, 18:1 n-9, 16:1 n-9 (0.58)

^1^R2Y implies the squared Pearson correlation coefficient calculated between modelled and measured values for the lipoprotein features.

^2^Q2Y implies the squared Pearson correlation coefficient calculated between predicted and measured values for the lipoprotein features.

^3^RMSEP implies the root mean square error of prediction error (Martens and Dardenne 1998) estimated from repeated double cross validation (Westerhuis et al. 2008).

^4^Pearson´s correlation coefficient estimated from the raw data, see suppl. material. 2B.
